# Synaptophysin accelerates synaptic vesicle fusion by expanding the membrane upon neurotransmitter loading

**DOI:** 10.1126/sciadv.ads4661

**Published:** 2025-04-23

**Authors:** Julia Preobraschenski, Alex J. B. Kreutzberger, Marcelo Ganzella, Agnieszka Münster-Wandowski, Mark A. B. Kreutzberger, Linda H. M. Olsthoorn, Sascha Seibert, Volker Kiessling, Dietmar Riedel, Agata Witkowska, Gudrun Ahnert-Hilger, Lukas K. Tamm, Reinhard Jahn

**Affiliations:** ^1^ Laboratory of Neurobiology, Max-Planck-Institute for Multidisciplinary Sciences, Göttingen 37077, Germany.; ^2^ Institute for Auditory Neuroscience, University Medical Center, Göttingen 37075, Germany.; ^3^ Multiscale Bioimaging Cluster of Excellence (MBExC), University of Goettingen, Göttingen 37075, Germany.; ^4^ Department of Molecular Physiology and Biological Physics, University of Virginia, Charlottesville 22903, VA, USA.; ^5^ Center for Membrane and Cell Physiology, University of Virginia, Charlottesville 22903, VA, USA.; ^6^ Institute für Integrative Neuroanatomy, Charité – Universitätsmedizin Berlin, Berlin 10117, Germany.; ^7^ Department of Biochemistry and Molecular Genetics, University of Virginia School of Medicine, Charlottesville 22903, VA 22903, USA.; ^8^ Facility for Transmission Electron Microscopy, Max-Planck-Institute for Multidisciplinary Sciences, Göttingen 37077, Germany.

## Abstract

Synaptic transmission is mediated by the exocytotic release of neurotransmitters stored in synaptic vesicles (SVs). SVs filled with neurotransmitters preferentially undergo exocytosis, but it is unclear how this is achieved. Here, we show that during transmitter loading, SVs substantially increase in size, which is reversible and requires synaptophysin, an abundant membrane protein with an unclear function. SVs are larger when synaptophysin is knocked out, and conversely, liposomes are smaller when reconstituted with synaptophysin. Moreover, transmitter loading of SVs accelerates fusion in vitro, which is abolished when synaptophysin is lacking despite near normal transmitter uptake. We conclude that synaptophysin functions as a curvature-promoting entity in the SV membrane, allowing for major lateral expansion of the SV membrane during neurotransmitter filling, thus increasing their propensity for exocytosis.

## Teaser

Synaptophysin allows synaptic vesicles to expand during loading with neurotransmitters, resulting in faster fusion.

## INTRODUCTION

Synaptic transmission is mediated by neurotransmitters (NTs) that are released from synaptic vesicles (SVs) by Ca^2+^-triggered exocytosis. Thereafter, empty SVs are retrieved by endocytosis and need to be refilled for another round of release. For reliable signaling, synapses need to ensure that only SVs filled with NTs undergo exocytosis. Filled SVs exhibit a higher release probability than empty SVs (*1*–*3*). However, it is not known how filled SVs are distinguished from empty SVs and why they are preferred for calcium-dependent exocytosis.

Several studies suggest that empty SVs are smaller than filled SVs such as cholinergic SVs of the marine ray Torpedo (*4*), aminergic vesicles from leech neurons (*5*), and glutamatergic vesicles in *Drosophila* (*6*, *7*). Similarly, studies on isolated SVs suggest that SVs expand during NT filling, with up to a doubling in volume being reported (*8*, *9*). Considering that after endocytosis, SVs need to acquire hundreds of millimolar of NTs within seconds from the surrounding cytoplasm, it is conceivable that the increase in SV size is driven by an increase in osmotic pressure associated with transmitter loading (*5*, *7*). However, how can vesicles expand in size?

One possibility is that empty SVs are collapsed or crumpled and merely expand into a round vesicle upon filling. However, SVs, with a diameter of only 42 nm in mammals, belong to the smallest known intracellular membrane vesicles, with a membrane curvature at the limit of what is possible for bilayers, and there is no evidence suggesting that empty vesicles are not spherical. On the other hand, the elasticity of lipid membranes is limited, i.e., they can only be stretched by ~2 to 3% in area without rupture (*10*–*13*). Thus, the substantial size increases that appear to be associated with NT loading (*8*) cannot be reconciled with the known physical properties of lipid bilayers.

The question then arises whether proteins may contribute to vesicle swelling. Budzinski *et al.* (*8*) suggested that the vesicle protein SV2, a multispanning transmembrane protein of unclear function, may contribute to vesicle swelling, but it remained unclear how this is achieved. To explore this question, using purified SVs from mammalian brain, we now show that SVs undergo a substantial size increase upon glutamate (Glut) accumulation that is reversible upon Glut efflux, suggesting that the SV membrane is capable of elastic expansion. In addition, using an established in vitro system for monitoring Ca^2+^-dependent fusion of SVs with reconstituted planar supported membranes, we found that filled SVs fuse faster and more efficiently than empty SVs. Both loading-dependent swelling and increase in fusion propensity were dependent on the presence of synaptophysin, a major SV protein of hitherto unknown function. Moreover, in reconstituted membranes, synaptophysin increases membrane curvature, resulting in smaller vesicles. We conclude that synaptophysin, probably in conjunction with other multispanning SV proteins, functions as an “elastomer” that contracts vesicles when empty but allows for substantial lateral expansion of the SV membrane during NT loading.

## RESULTS

### SVs increase in size upon loading with NTs

**Fig. 1.**
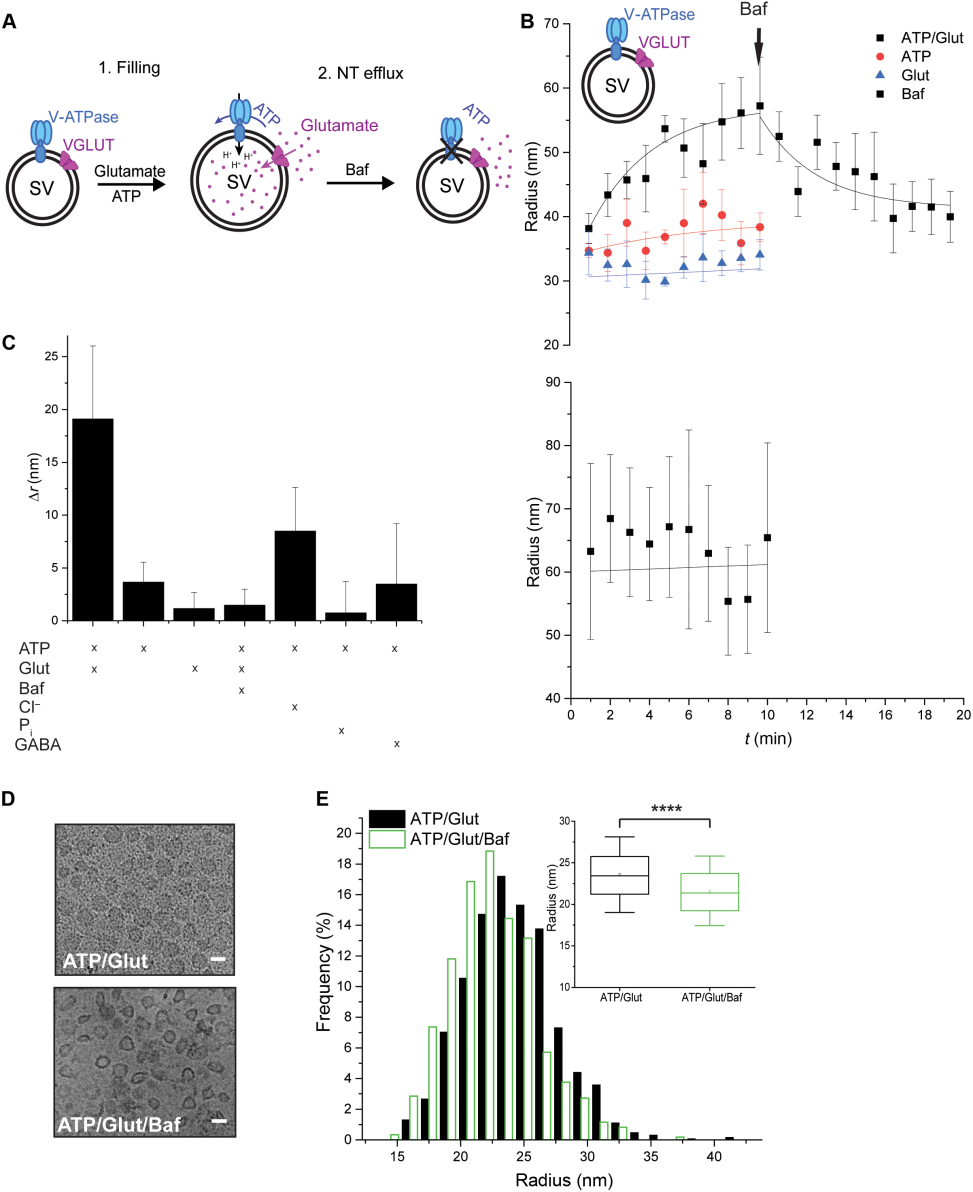
SVs reversibly expand upon Glut loading. (**A**) Cartoon illustrating the SV size changes associated with Glut influx and efflux. (**B**) Time-dependent size changes, measured by DLS, of SVs (upper panel) and proteoliposomes reconstituted with VGLUT1 and TF_o_F_1_ (lower panel) upon incubation in 10 mM K-glutamate, 4 mM Mg-ATP, and 4 mM KCl (Glut uptake conditions: ATP/Glut; black line), 4 mM ATP (red line), and 10 mM K-glutamate (Glut; blue line) in 300 mM glycine and 5 mM Hepes (pH 7.4; uptake buffer). The reaction was started by the addition of ATP and Glut. In addition, SV size changes under Glut uptake conditions after addition of Baf were monitored. The time point of addition (10 min) is marked with an arrow. (**C**) Increase in SV radii, measured by DLS, in the presence of various substrates after an incubation of 10 min at 37°C. (**D** and **E**) Representative micrographs of SVs acquired by cryo-EM (scale bars correspond to 50 nm) (D) and histogram and box plot showing the SV diameter determined by cryo-EM (E) under Glut uptake conditions (ATP/Glut; black) and in the presence of Glut ATP and Baf (ATP/Glut/Baf; green). The box in the box plot represents data between 25 and 75%, and the whiskers show data between 10 and 90%. The dot within the box indicates the mean value, and the line indicates the median. The data in the box plot were analyzed using a two-tailed paired *t* test, ****P < 0.0001. [(B) and (C)] Measured by DLS. (B) *n* = 3 to 6; (C) *n* = 2 to 6; (D) *n* = 2; number of SVs, 602 (ATP/Glut/Baf) and 948 (ATP/Glut).

We used dynamic light scattering (DLS) to monitor time-dependent size changes of isolated rat brain SVs during Glut uptake under standard conditions (Fig. 1, B and C) (*14*). A substantial increase in the average diameter was observed (~1.5-fold after 10 min), which was absent when either Glut or adenosine 5′-triphosphate (ATP) was omitted [Fig. 1B; see fig. S1 (B to D) for size distribution], in line with previous studies (*8*, *9*). The size increase scaled with the external Glut concentration, being maximal at 10 mM Glut (not shown) and completed within ~10 min (Fig. 1B), thus corresponding to Glut uptake as previously described (*15*, *16*). No notable size changes were observed when the vacuolar-type ATPase (V-ATPase) was inhibited by bafilomycin (Fig. 1C) or when gamma-aminobutyric acid (GABA) was used instead of Glut (Figs. 1C and fig. S1A). The latter is expected because the proportion of GABAergic SVs in our preparation is less than 20% (*17*). Other anions also did not result in major size changes, although a slight increase was observed in the presence of chloride ions that are known to be also transported by a vesicular glutamate transporter (VGLUT; Fig. 1C and fig. S1A) (*14*, *18*). A size increase of SVs after Glut loading, albeit less marked, was also observed when SVs were analyzed by cryo–electron microscopy (cryo-EM) after loading {Fig. 1, D and E; filled SVs (ATP/Glut) *r* = 23.58 nm; empty SVs [ATP/Glut/bafilomycin A1 (Baf)], *r* = 21.6 nm; note that the apparent diameters determined by DLS are larger because they include the hydrated protein coats}.

Next, we tested whether the size increase of SVs is reversible. To this end, we preloaded the SVs with Glut and then induced Glut efflux by abolishing the membrane electrochemical potential (Δμ*H*^*+*^) that is required for retaining Glut inside SVs (*19*–*21*). When the V-ATPase was blocked with Baf, the SV radius decreased over time, reaching almost the radius before loading (Fig. 1B and fig. S1E). Last, we investigated whether functionally active liposomes reconstituted with VGLUT1 and a bacterial F_o_F_1_-ATPase (*14*, *18*, *22*) undergo similar size changes upon Glut loading. This was not the case (Fig. 1B, lower panel) despite efficient uptake [not shown; (*14*)], suggesting that other SV membrane components must be responsible for the size increase.

**Fig. 2.**
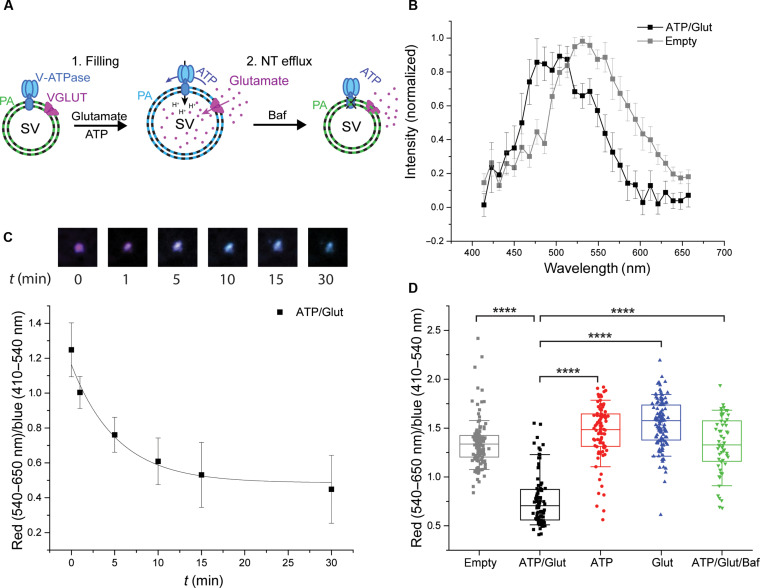
Size changes associated with Glut loading affect the lipid order of the SV membrane. (**A**) Illustration of changes in lipid order in empty and Glut-filled SVs. (**B**) Emission spectra of PA dye–labeled, Glut-filled (black squares) and empty (gray squares) SVs. (**C**) Time course of lipid order changes represented as red (540 to 650 nm)/blue (410 to 540 nm) ratios of PA dye–labeled SVs during a time course of Glut uptake (exposure to ATP/Glut at time 0) with representative corresponding images of a single SV. (**D**) Box plot representing red (540 to 650 nm)/blue (410 to 540 nm) ratios of PA dye–labeled SVs in the absence of substrates (empty; gray), under Glut-loaded conditions (ATP/Glut; black), and in the presence of ATP (red), Glut (blue), and ATP, Glut, and Baf (ATP/Glut/Baf; green). The box represents data between 25 and 75%, and the line in the box indicates the median. The whiskers show data between 10 and 90%. Individual values are displayed as gray (empty) and black rectangles (ATP/Glut), red circles (ATP), and blue upward-facing (Glut) and green downward-facing (ATP/Glut/Baf) triangles. The data in the box plot were analyzed using a two-tailed paired *t* test. ****P < 0.0001. (B) *n* = 7; (C) *n* = 7; (D) *n* = 55 to 128.

We then investigated whether the membrane lipids change their organization during vesicle swelling. To this end, we used a recently developed solvatochromic fluorescent probe [push-pull pyrene dye (3,8-dibutyl-6-(piperidin-1-yl)pyrene-1-carbaldehyde, PA)] that inserts into membranes and responds with a shift in the emission spectrum during changes of the polarity and hydration at the membrane interface (*23*) and recorded spectral changes of single SVs using a confocal microscope. A blue shift was observed in vesicles loaded with Glut (ATP/Glut) (Fig. 2) with a loading time course (Fig. 2C) of the same order as in vitro Glut uptake and SV size increase, indicating substantial changes in membrane hydration and lipid packing (to a more ordered state) upon NT filling.

Together, our data extend previous observations (*8*) showing that SVs undergo substantial swelling upon Glut loading, which is reversible upon Glut efflux and associated with a change in lipid order. The degree of expansion is difficult to reconcile with the limited ability of lipid bilayers to be stretched without rupture, raising the question whether SV membrane proteins are responsible for the remarkable elasticity of the SV membrane.

### Synaptophysin increases the membrane elasticity and spontaneous curvature of SVs

SVs belong to the best studied trafficking vesicles, and both their resident proteins and membrane lipids were extensively characterized (*24*, *25*). In addition to the proteins responsible for membrane fusion [soluble *N*-ethylmaleimide–sensitive factor attachment protein (SNAP) receptors (SNAREs) and synaptotagmins] and NT loading (vesicular NT transporters and V-ATPase), SVs have several multispanning transmembrane proteins of unclear function that are present on virtually all SVs. Synaptophysin (mainly its major isoform synaptophysin I) is the most abundant among them, with on average 30 copies per SV (*24*). Synaptophysin has four transmembrane domains (TMDs), with both the C and N termini exposed to the cytoplasm, and two intravesicular loops, one of which is glycosylated. In addition, SVs contain isoforms of two more tetraspan protein families, termed synaptogyrin (*26*) and secretory carrier membrane protein (*27*), that are less abundant and, in contrast to the largely neuron-specific synaptophysin, ubiquitously expressed. Despite their conservation between vertebrates and invertebrates, none of these proteins appears to be essential as deletion of some or all of them in mice or *Caenorhabditis elegans* (*28*–*31*) did not result in any major synaptic or developmental phenotype (*30*, *31*).

**Fig. 3.**
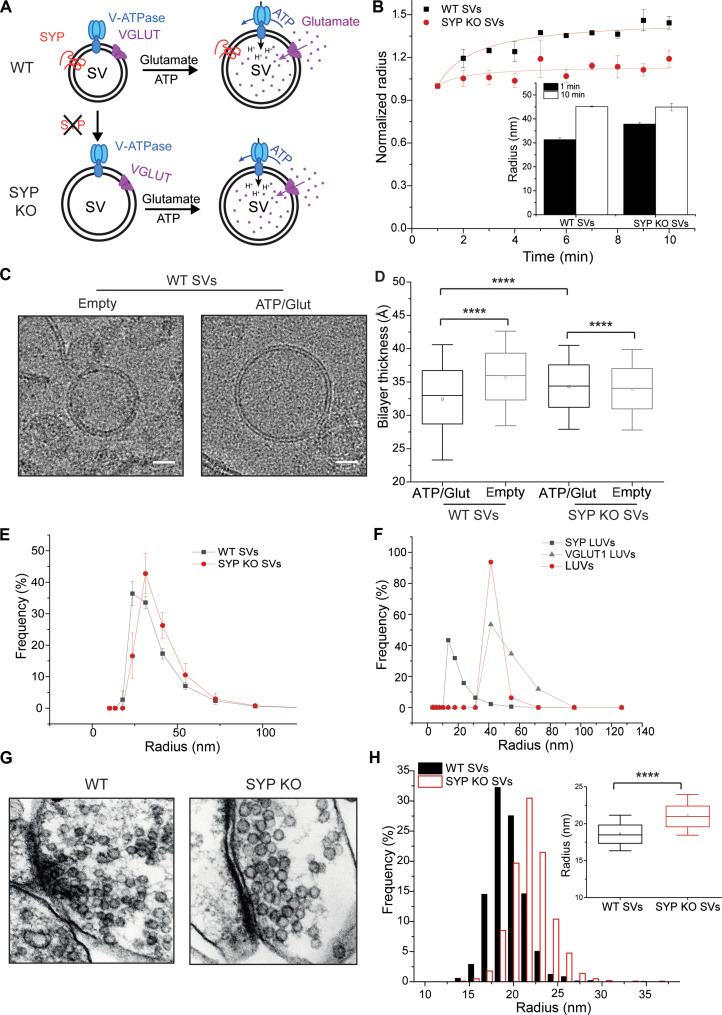
SYP KO SVs expand less and are larger than WT SVs. (**A**) Schematic depicting Glut filling–associated size changes in WT and SYP KO SVs. (**B**) Time-dependent expansion of WT (black line) and SYP KO (dark red line) SVs under Glut uptake conditions. Inset: radii of WT and SYP KO SVs after 1-min (black bars) and 10-min (white bars) incubation under Glut uptake conditions. (**C**) Representative micrographs of empty and filled WT SVs acquired by cryo-EM. Scale bars correspond to 20 nm. (**D**) Bilayer thickness of empty and Glut-filled WT and SYP KO SVs. (**E**) Size distribution of isolated WT (black line) and SYP KO (dark red line) mouse SVs analyzed by DLS. (**F**) Size distribution of synaptophysin-reconstituted (SYP LUVs), VGLUT1-reconstituted (VGLUT1 LUVs), and protein-free (LUVs) liposomes demonstrating that synaptophysin supports the formation of liposomes with a high curvature. (**G**) Representative micrographs of SVs in mouse hippocampal WT (left panel) and SYP KO (right panel) neurons acquired by electron microscopy. (**H**) Histogram and box plot (inset) showing the size distribution of WT (black bars and box) and SYP KO (dark red bars and box) SVs in mouse hippocampal neurons imaged and analyzed using electron microscopy. The box in the box plots in (D) and (H) represents data between 25 and 75%, and the whiskers show data between 10 and 90%. The dot within the box indicates the mean value, and the line indicates the median. The data in the box plots in (D) and (H) were analyzed using a two-tailed paired *t* test. ****P < 0.0001. [(A) to (D)] Analyzed by DLS. (B) *n* = 2 to 3; (D) 15,829 to 38,238 sites in 15 to 29 SVs per condition. (E) *n* = 3; (F) *n* = 3; (H) *n* = 7 to 9; number of SVs, 1383 (WT) and 1011 (SYP KO).

Synaptophysin was shown to bind cholesterol [reviewed in (*32*)], raising the possibility that it may influence protein-lipid interactions in the vesicle membrane. We therefore examined whether synaptophysin I plays a role in the SV size changes during NT uptake. To this end, we isolated SVs from transgenic mice lacking synaptophysin I [synaptophysin knockout (SYP KO) SVs] and measured the diameter changes associated with Glut filling using DLS (Fig. 3, A and B, and fig. S2, A and B). Notably, during Glut uptake, the diameter of SYP KO SVs increased substantially less (~1.19-fold) than that of SVs isolated in parallel from wild-type (WT) littermates (WT SVs; ~1.44-fold) (Fig. 3, A and B, and fig. S2, A and B), suggesting that synaptophysin I affects the membrane elasticity of SVs. In addition, we found that in WT SVs, the size increase was associated with substantial lipid bilayer thinning upon loading with NTs (empty SVs, 3.6 nm; filled SVs, 3.2 nm; Fig. 3, C and D, and fig. S2C), whereas in SYP KO SVs, the bilayer was overall thinner (empty SVs, 3.4 nm) and did not change upon filling (filled SVs, 3.4 nm) (Fig. 3D).

Notably, the radius of empty SYP KO SVs (*r* = 37.82 nm) was larger than that of WT SVs (*r* = 31.32 nm) (Fig. 3E), in line with recent work analyzing synaptophysin and synaptogyrin family quadruple KO SVs in the mouse brain (*33*). After Glut loading, however, the radius (SYP KO, *r* = 44.96 nm) was similar to that of WT SVs (*r* = 45.17 nm) (Fig. 3B, inset). In line with this finding, only a slight elevation of uptake in SYP KO in contrast to WT SVs was observed (fig. S2D), in agreement with previous reports showing unchanged or slightly enhanced quantal content in KO neurons (*29*, *31*). Thus, it appears that synaptophysin increases the spontaneous curvature of membranes, resulting in smaller vesicles, similar to synaptogyrin (*34*). To test this idea, we purified synaptophysin from rat brain (fig. S3A) and reconstituted it into both large (fig. S3B) and giant unilamellar liposomes (LUVs and GUVs, respectively). Reconstitution in LUVs resulted in liposomes that were markedly smaller than liposomes devoid of proteins or reconstituted with equal amounts of VGLUT, another multispanning resident of SVs (Fig. 3F). Reconstitution of synaptophysin in GUVs resulted in GUVs that were more deformed than empty GUVs, frequently associated with a flattening of the vesicle, with synaptophysin accumulating in the highly curved rim region (fig. S3C). Last, we carefully quantified the size distribution of SVs in primary hippocampal neuronal cultures from WT and SYP KO mice (Fig. 3, G and H, and fig. S3D) using transmission electron microscopy and found that the SYP KO SVs (*r* = 21.02 ± 0.6 nm) were significantly larger than WT SVs (*r* = 18.57 ± 0.5 nm) (Fig. 3H, inset), confirming the size difference between the populations that we observed after vesicle isolation (Fig. 3E). Beyond that, the number of SVs in SYP KO synapses was reduced, albeit not to a significant level, despite the synapse area remaining similar (fig. S3, E and F). Together, these data establish synaptophysin as a major regulator of membrane curvature and membrane elasticity in the SV membrane.

### Loading-dependent size increase enhances the fusion propensity of SVs

The data discussed so far show that during loading of SVs with NTs, there is a considerable increase in size that depends on the presence of synaptophysin. One possible interpretation of this observation is that loading is associated with an increase in osmotic pressure, which increases membrane tension, but it depends on the membrane elasticity whether increased tension leads to an expansion of the membrane area. Area expansion, on the other hand, will increase surface hydrophobicity because the phospholipid head groups are spaced farther apart. Increased hydrophobicity is expected to lower the energy barrier for exocytotic membrane fusion, which may explain why filled vesicles are preferred for exocytosis (see Introduction).

**Fig. 4.**
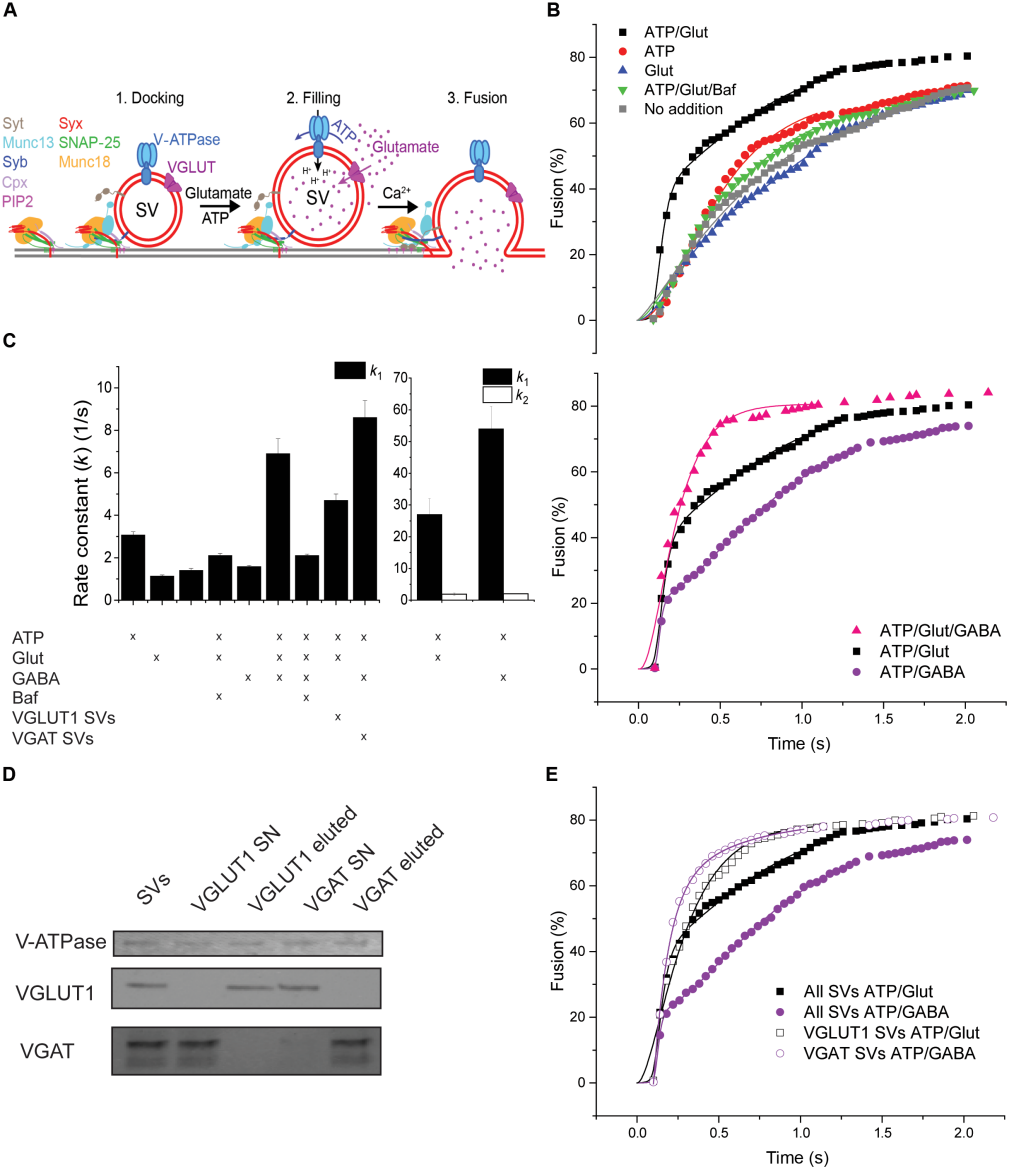
NT-filled SVs fuse faster than empty SVs. (**A**) Cartoon of the fusion assay of SVs [see (*35*) for details]. Planar supported bilayers containing the SNARE proteins syntaxin-1A and SNAP-25 were incubated with SVs in the presence of complexin-1, Munc18, and Munc13. Subsequently, ATP and NTs were added as indicated to allow for SV filling for 20 min. Ca^2+^ (100 μM) was then added to trigger rapid fusion of SVs, while images of single vesicles were acquired on a total internal reflection fluorescence microscope. (**B**) Cumulative distribution of the fusion delay times of predocked SVs from the time of Ca^2+^ addition (shown as the percent of docked vesicles at the onset of the reaction). Note that the data for ATP/Glut are shown in both panels for comparison. (**C**) Rate constants of whole-brain SV fusion calculated by fitting using either one-component (*k*_1_; left panel) or two-component first-order kinetic models (*k*_1_ and *k*_2_) (see Materials and Methods for details). (**D**) Immunoblot showing the enrichment of VGLUT1 and VGAT, respectively, in fractions obtained by immunoisolation using beads coupled with VGLUT1- and VGAT-specific antibodies. Compared to the starting vesicle fraction (SV), the supernatants from the immunoisolation experiments (SN) reveal selective depletion of the respective vesicle populations. For reference, a blot for the V0a1 subunit of the V-ATPase is shown, which is present on all SVs. (**E**) Comparison between the fusion kinetics of immunoisolated glutamatergic and GABAergic SVs with those of nondepleted SVs after preloading with the respective NT (all SVs ATP/Glut and all SVs ATP/GABA versus VGLUT1 SVs ATP/Glut and VGAT SVs ATP/GABA). (C) *n* = 4 to 7. Note that both glutamatergic and GABAergic vesicles displayed similar fast fusion kinetics when incubated with ATP and the respective NT. Open symbols: experiments with immunoisolated SVs; closed symbols: experiments with conventionally isolated SVs.

For these reasons, we tested whether SVs that are loaded with transmitters have a higher ability to fuse than unloaded SVs, and if this is the case, whether such an increase depends on the presence of synaptophysin. To this end, we used a recently developed in vitro assay for measuring calcium-dependent fusion with a single vesicle resolution, which recapitulates major aspects of synaptic exocytosis (*35*). In this assay, SVs are first docked to a planar supported membrane containing the SNAREs syntaxin-1 and SNAP-25 and the SNARE regulatory proteins complexin-1, Munc18, and Munc13, and then fusion is initiated by addition of Ca^2+^ (Fig. 4A).

Using this approach, we first analyzed whether fusion is related to NT uptake. We found that SVs loaded with Glut fuse substantially faster than SVs in which Glut loading was prevented by omitting Glut or ATP or by including the V-ATPase inhibitor BafA [Fig. 4, B (upper panel) and C, and fig. S4]. Kinetic analysis of the delay time between triggering exocytosis by calcium and fusion of SVs preloaded with Glut revealed a fast component and a slow component (*k*_1_ = 27 s^−1^, 70%; *k*_2_ = 1.9 s^−1^, 30%; Fig. 4C and fig. S3A). As the assayed sample consisted of whole-brain SVs, we hypothesized that the slow component may be due to the presence of nonglutamatergic vesicles that are mostly GABAergic and make up ~20% of the population (*17*). To test this hypothesis, we compared the fusion kinetics of SVs preloaded with Glut, GABA, or both NTs together (Fig. 4, B and C, and fig. S4). We observed a biexponential response upon preloading with either Glut or GABA [Fig. 4, B (lower panel) and C], but in the case of GABA, the slow component was larger (*k*_2_ = 2.02 s^−1^, 80%) than the fast component (*k*_1_ = 54 s^−1^, 20%). When we preloaded SVs with both NTs, the fusion kinetics displayed only the fast component (*k*_1_ = 6.9 s^−1^; Fig. 4, B and C, and fig. S4). Intriguingly, the overall fusion efficiency was unchanged under all conditions, with ~80% of all docked vesicles being fused at the end of the experiment.

Together, these data strongly support the hypothesis that the fusion of SVs is accelerated when they are filled with NTs and that this acceleration does not depend on the type of NT. To further corroborate these findings, we enriched glutamatergic or GABAergic SVs by immunodepletion from the starting mixed SV population (Fig. 4D). As expected, SV enrichment removed the slow component for both glutamatergic and GABAergic SVs (Fig. 4E). Thus, glutamatergic and GABAergic SVs, when filled with their respective NT, exhibited exclusively fast fusion kinetics and no slow-fusing population (Fig. 4, B and E). The slow-fusing components in the mixed populations with only one substrate added came from those vesicles that were not loaded.

**Fig. 5.**
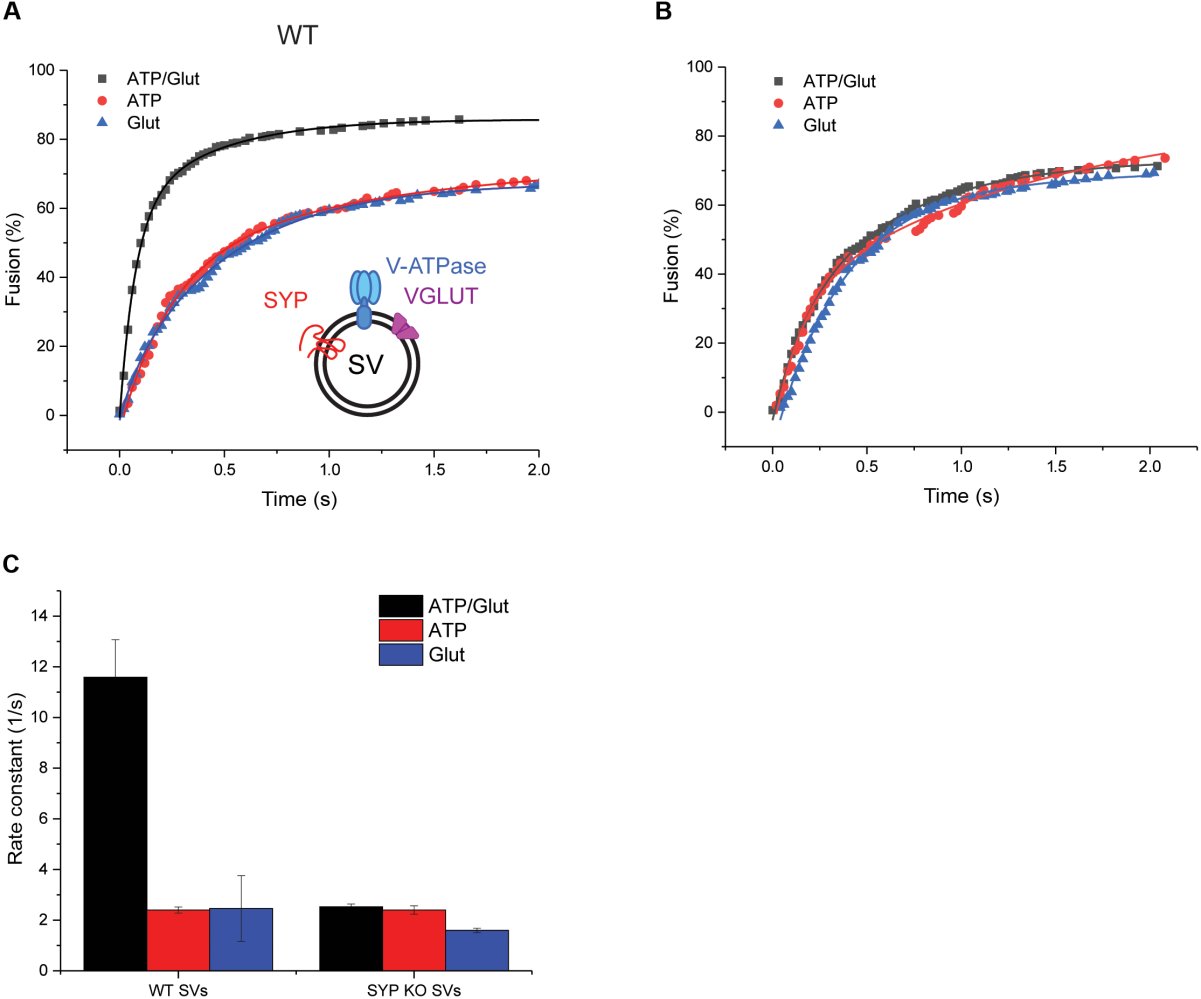
In contrast to SVs from WT mice, fusion of SVs from SYP KO mice is not enhanced upon loading with Glut. Fusion of WT (**A**) and SYP KO (**B**) SVs after Glut preloading (ATP/Glut; black squares) in comparison to control conditions [ATP only (ATP; red circles) and Glut only (Glut; blue triangles)]. (**C**) Rate constants of WT and SYP KO SVs under uptake and control conditions. See Fig. 4 for details of the assay. (C) *n* = 4 to 9. Note that in these experiments, the two kinetic components could not be clearly resolved anymore, possibly due to differences in the vesicle preparation methods (mouse versus rat).

In a final set of experiments, we asked whether the acceleration of fusion upon transmitter filling depends on the presence of synaptophysin. To this end, SVs were prepared from WT and SYP KO mice and tested for their change in fusion activity upon preloading with Glut. Whereas Glut-preloaded SVs from WT mice fused faster than empty SVs (Fig. 5, A and C), Glut-preloaded SVs from SYP KO mice did not show any notable acceleration of fusion when compared to empty SVs (Fig. 5, B and C), even though there was no difference in Glut loading (fig. S2D).

## DISCUSSION

In the present study, we demonstrate that synaptophysin, a highly abundant and evolutionarily conserved membrane protein of hitherto unknown function, is a key player in the reversible expansion of SVs that occurs during loading with NTs. Moreover, we show that synaptophysin-dependent expansion facilitates Ca^2+^-triggered membrane fusion of SVs.

The size increases observed here and in previous studies (*8*, *9*) are substantial, with the observed diameter increases ranging from 10 to 50% depending on the method. Note that the size increases reported by DLS, when compared to cryo-EM, may be overestimated because even a minor degree of cluster/aggregate formation (which was not evident in the EM analysis) will disproportionally increase the radius estimates. However, all increases were completely reversible upon inducing Glut efflux, which is unlikely if aggregates form by hydrophobic interactions. In any case, our findings imply that the SV membrane can be stretched considerably without rupture or leakage, which is associated with substantial thinning of the membrane. How does synaptophysin, and perhaps also the previously invoked protein SV2, change the properties of the SV membrane in such a marked fashion? One possibility may be that synaptophysin and perhaps also SV2 are laterally stretchable and thus convey elasticity to the membrane. Both synaptophysin and SV2 are highly abundant, with 30 and 5 to 15 copies per SV, respectively (*24*, *36*). Considering that synaptophysin contains 4 TMDs and SV2 contains 12 TMDs, the proteins together contribute ~240 TMDs per SV, probably even more when including the less abundant isoforms of the synaptophysin family (*37*, *38*). Even when tightly packed, these TMDs would occupy a substantial share of the SV membrane volume [at least 10%; see (*24*)]. While, for synaptophysin, only a low-resolution hexameric cryo-EM model is available (*39*), a high-resolution nuclear magnetic resonance structure for its relative synaptogyrin (which is a stable monomer) has recently been published (*34*). Synaptogyrin’s TMDs form two pairs of parallel alpha helices that are tilted with respect to each other, and the AlphaFold prediction for the structure of the TMDs of synaptophysin is very similar. It is conceivable that the TMDs are becoming more tilted and partially pulled apart upon stretching, creating shallow grooves that would need to be filled with membrane lipids.

Alternatively, it is conceivable that synaptophysin and its relatives alter the properties and/or composition of the membrane lipids. Intriguingly, the SV membrane is rich in cholesterol (close to 40%) and, even more remarkable, contains an unusually high share of polyunsaturated phospholipid side chains (~70%) (*24*) that were shown to soften the membrane, making it less resistant to curvature changes. Molecular dynamics simulations suggest a high degree of conformational dynamics including partial back-flipping of unsaturated side chains (*40*, *41*). Synaptophysin and its relatives may increase distortion of the surrounding lipids, e.g., by hydrophobic mismatch as shown previously for rhomboid proteins (*42*), which would facilitate side-chain flipping. Assisted by cholesterol redistribution, such back-flipping may stabilize the membrane after stretching/thinning, thus preventing leakage and membrane rupture. Synaptophysin, perhaps via specifically bound cholesterol (*32*), may preferentially bind to highly unsaturated phospholipids, thus creating a nanodomain enriched in these lipids that is delivered to SVs during biogenesis and may remain associated with the protein during exo-endocytotic cycling.

After endocytosis, SVs are reloaded within minutes with hundreds of millimolar of NTs. The associated increase in osmotic pressure is probably substantial (*14*) and likely to provide the driving force for vesicle expansion. We observed an increase in surface hydrophobicity during expansion, which is expected because the polar phospholipid head groups are spaced farther apart. Increased surface hydrophobicity reduces electrostatic repulsion between membranes and is known to reduce the energy barrier for membrane fusion (*43*), thus explaining why transmitter-filled SVs exhibit a higher release probability (*1*, *2*).

The biological significance of the reversible elastic expansion of SVs during loading remains to be established. Neither synaptophysin nor SV2 isoforms are essential for survival. Their deletion in mice results in morphologically largely normal animals that exhibit synaptic and behavioral defects ranging between barely detectable and moderate (*28*, *29*, *44*, *45*). The reduced elasticity of the SV membrane in SYP KO mice may be offset by the larger vesicle size [which was also observed in *Drosophila* deletion mutants of the related protein synaptogyrin (*46*) and in mouse brain SVs lacking synaptophysin I, synaptoporin, synaptogyrin 1, and synaptogyrin 3 (*33*)], explaining why KO vesicles, despite not being capable of expanding, can be loaded with comparable amounts of NT. Intriguingly, in mice lacking synaptophysins and synaptogyrins, the release probability, at least in some synapses, was increased rather than decreased (*31*), in contrast to our in vitro observations. However, defects in SV function may be compensated by other mechanisms. For instance, these proteins are known to influence sorting of the SNARE synaptobrevin [synaptophysin; (*29*)], the Ca^2+^-sensor synaptotagmin (SV2), and probably also the V-ATPase, which may contribute to the KO phenotypes [see, e.g., (*31*, *47*) for a more detailed discussion]. Also, it remains to be established whether empty or incompletely filled vesicles also exhibit a lower release probability when synaptophysin is lacking.

In summary, our data show that synaptophysin is responsible for conveying high elasticity to the SV membrane, allowing for its reversible expansion when stretched by osmotic pressure. They also confirm and extend previous observations showing that synaptophysin and its family member synaptogyrin sense/induce curvature and thus are also responsible for the uniformly small size of SVs (*34*, *46*, *48*). It is conceivable that not only the synaptophysin and SV2 protein families but also other structurally similar tetraspanin proteins such as those grouped together in the “MARVEL” family (*49*) may function as organizers of membrane lipid nanodomains. Moreover, it is possible that the stretched membrane is stabilized by adsorbed amphiphilic proteins, which is known to be facilitated by unsaturated membrane lipids (*40*), possibly including the vesicular phosphoprotein synapsin that is known to be highly surface active (*50*).

## MATERIALS AND METHODS

### SV isolation

#### Animals

Adult Wistar rats were purchased from Charles River Laboratories or Janvier, and SYP KO and WT control mice were provided by R. Leube (RWTH Aachen, Germany) and were kept until use at a 12:12-hour light/dark cycle with food and water ad libitum. The facility is registered according to §11 Abs. 1 TierSchG (Tierschutzgesetz der Bundesrepublik Deutschland, Animal Welfare Law of the Federal Republic of Germany), as documented by 33.23-42508-066-§11, dated 16 November 2023 (“Erlaubnis zum Halten von Wirbeltieren zur Versuchszwecken,” “Permission to keep vertebrates for experimental purposes”), by the Niedersächsisches Landesamt für Verbraucherschutz und Lebensmittelsicherheit (Lower Saxony State Office for Consumer Protection and Food Safety).

#### SVs

Rat SVs were isolated from whole rat brain according to previous publications (*24*, *51*, *52*). Briefly, an SV-enriched fraction, LP2, was prepared by differential centrifugation and centrifuged on a continuous sucrose density gradient. The gradient fraction enriched in SVs (0.04 to 0.4 M sucrose) was collected and separated by size exclusion chromatography using controlled-pore glass beads.

Whole-brain mouse SVs were prepared following a recently published protocol (*53*). Here, the SV-enriched LS1 fraction (one step before obtaining LP2) was loaded on a two-step sucrose gradient (0.3 and 0.7 M sucrose) and subjected to high-speed centrifugation. Afterward, 12 of the top fractions were discarded and 8 of the bottom fractions enriched in SVs including the pellet were collected and further purified by size exclusion chromatography on controlled-pore glass beads, as described. The peak fractions containing SVs were directly used for DLS measurements on the same day without pelleting to avoid aggregation of SVs. For the experiments involving pyrene dye labeling and measurement of single-vesicle fusion, the SVs were pelleted by ultracentrifugation, resuspended in 320 mM sucrose and 5 mM Hepes (pH 7.4), flash frozen, and stored at −80°C until use. For cryo-EM, the pelleted SVs were resuspended in 300 mM glycine and 5 mM Hepes (pH 7.4) and used on the same day for sample preparation without prior freezing. The protein concentration range of the pelleted SVs varied from 1.5 to 2.5 mg/ml. For the comparison of WT and SYP KO SVs, age-matched (10 to 12 weeks) homozygous animals of mixed sexes were used. The WT SVs were derived from littermates.

### DLS measurements

DLS measurements of SVs and proteoliposomes were performed using a DynaPro Titan (Wyatt Technology) instrument. The particle count was adjusted to ~120,000 at a laser power of 5% to ensure comparable measurement conditions. To assess NT uptake–dependent size changes, measurements were performed at 37°C in in 300 mM glycine and 5 mM Hepes (pH 7.4; uptake buffer). Depending on the experiment, the following additions were made as indicated: 10 mM K-glutamate, 10 mM KCl, 10 mM K-phosphate or 10 mM GABA (unless indicated otherwise), 4 mM Mg-ATP, and 4 mM KCl for Glut uptake and 10 mM KCl for GABA uptake. Baf was used at a concentration of 0.5 μM. To determine the size distribution of SVs and proteoliposomes, the samples were measured at 25°C in 300 mM glycine and 5 mM Hepes (SVs) or reconstitution buffer (proteoliposomes).

Time-dependent SV size changes under various conditions were monitored by measuring each sample one time per minute for 10 or 20 (measurements with Baf) consecutive minutes. One measurement consisted of 10 acquisitions with an acquisition time window of 6 s. For every SV preparation, all measurements were repeated at least two times per condition. For end-point measurements, each sample was measured three times. Each measurement consisted of 20 acquisitions using an acquisition time window of 5 s.

### Cryo-EM of isolated SVs

#### Measurement of diameter

SVs were vitrified using a Vitrobot Mark IV (FEI) at 30°C and 97% humidity by applying the sample on a glow-discharged “holey” carbon foil (quantifoil grid), blotting twice for 1 s at a “blot-force” of 2, and subsequently plunging frozen in liquid ethane. Cryo-EM was performed using a Titan Krios microscope equipped with a Falcon 3 camera (FEI, Hillsboro, OR) operating at 300 kV, running in twofold binning mode. Per condition, 554 to 949 SVs were analyzed, with the radius being determined by taking the average of the shortest and longest diameters of the SVs measured from a bilayer surface to bilayer surface.

#### Measurement and analysis of lipid bilayer thickness

For each condition, Lacey carbon grids with 300-mesh copper were prepared for freezing with an Electron Microscopy Sciences GloQube glow discharge system. Grids were prepared in one of two ways: (i) They were glow discharged using a positive voltage in the air chamber of the GloQube system, resulting in a negative surface. Subsequently, a thin film of graphene oxide was applied to the carbon face of the grid. (ii) The grids were glow discharged with a negative voltage in the air chamber of the GloQube system (positive surface). In this case, no graphene oxide film was applied.

Plunge-freezing was performed using a Vitrobot Mk IV. For each grid, 3 μl of sample was applied to the carbon side of the grid and then blotted with a force setting of 5 for 5 s. This was followed by immediate plunging of the grid into liquid ethane. The grids were then imaged on a Thermo Fisher Scientific Glacios TEM at 200 keV equipped with a Falcon 4 direct electron detector. The raw pixel size of each micrograph was 1.5 Å per pixel. Movies were motion corrected in cryoSPARC (*54*). For easier visualization and analysis, the motion-corrected movies were low-pass filtered in Spider (*55*).

Electron micrographs of SVs were analyzed following the procedure by Haeberle *et al.* (*56*) and using custom-made software written in Matlab (MathWorks, Natick, MA). Electron density profiles are extracted along the membrane of a selected vesicle in 1-pixel intervals and aligned relative to each other according to the characteristic profile (fig. S2C) (*56*). After running a moving average over 29 pixels corresponding to 49.3 Å, the positions of the two minima in the profiles of all segments are determined by fitting Gaussian curves to the data. The distances between the intensity minima of each segment are saved as bilayer thickness and reported in Fig. 3D. Thicknesses of 16,000 to 38,000 from 15 to 29 imaged SVs for each condition were determined.

### Protein purification

#### Synaptophysin

Native synaptophysin was purified from whole rat brain as described (*57*), except that 1% Na-cholate was used for solubilization of synaptophysin. Briefly, a solubilized synaptophysin-containing fraction was prepared by initial differential centrifugation followed by solubilization in 150 mM NaCl, 10 mM Na-phosphate (pH 7.2), 0.1 mM phenylmethylsulfonyl fluoride, pepstatin (2 μg/ml), and 1% (w/v) Na-cholate (Sigma-Aldrich) and purified using a monoclonal synaptophysin antibody (Cl 7.2, 101011, Synaptic Systems)–coupled resin (AminoLink Resin, Thermo Fisher Scientific). The protein was flash frozen in small aliquots and stored at −80°C until use.

#### VGLUT1

VGLUT1 was expressed in insect cells using the baculovirus expression system and purified as previously described (*14*, *22*). Briefly, streptavidin binding peptide–tagged *Mus musculus* VGLUT1 was expressed in *Trichoplusia ni* cells (High5, Invitrogen); solubilized in 300 mM KCl, 40 mM Tris (pH 7.3), 2 mM EDTA, 1× protease inhibitor cocktail (EDTA free, Merck), 5 mM beta-mercaptoethanol, and 1% dodecyl-beta-d-maltoside (Glycon); and purified by affinity purification using streptavidin beads (Pierce). After elution with 100 mM KCl, 15 mM Tris (pH 7.3), 0.6 mM EDTA, 2 mM (+)-biotin, 5 mM beta-mercaptoethanol, and 0.05% dodecyl-beta-d-maltoside and concentration, the protein was flash frozen and stored at −80°C until use.

#### SNARE protein, complexin-1, Munc18, and Munc13

Syntaxin-1a (residues 1 to 288) and quadruply dodecylated (through disulfide bonding of dodecyl methanethiosulfonate) SNAP-25 were purified as described in (*58*). Complexin-1 was purified as described in (*59*). Munc18 and Munc13 were purified as described in (*35*).

### Preparation and imaging of giant unilamellar vesicles containing synaptophysin

Fluorescently labeled GUVs reconstituted with synaptophysin were prepared from small unilamellar vesicles (SUVs) according to (*60*) with minor modifications. Briefly, SUVs containing synaptophysin were prepared by mixing lipids composed of 98:1:1 (mol %) 1,2-dioleoyl-*sn*-glycero-3-phosphocholine (DOPC, Avanti Polar Lipids):Texas Red 1,2-dihexadecanoyl-*sn*-glycero-3-phosphoethanolamine, triethylammonium salt (Texas Red DHPE, Thermo Fisher Scientific):1,2-dioleoyl-*sn*-glycero-3-phosphoethanolamine-*N*-(cap biotinyl) (sodium salt) (18:1 Biotinyl Cap PE, Avanti Polar Lipids) in 5% (w/v) Na-cholate (Merck) with synaptophysin at a molar protein:lipid ratio of 1:500 in 150 mM potassium chloride and 20 mM Hepes (pH 7.4). The mix was passed through a Sephadex G-25 column (Sigma-Aldrich) to remove the detergent. The resulting SUVs were vacuum dried on Pt electrodes to generate synaptophysin-containing GUVs by electroformation in 200 mM sucrose. The GUVs were then transferred to an imaging chamber containing a coverslip functionalized with biotinylated bovine serum albumin and neutravidin in 150 mM KCl, 1 mM MgCl_2_, and 20 mM Hepes (pH 7.4) and incubated for 30 min at room temperature to allow the GUVs to attach to the surface. Afterward, fluorescently labeled anti-synaptophysin antibody (synaptophysin 1-cyanine 2, 101011C2, Synaptic Systems) was added to the chamber at a final concentration of 1:700 and incubated for another 30 min at room temperature. Imaging was performed using a Zeiss LSM 780 confocal microscope using excitation at 488 nm and emission at 515 nm for monitoring synaptophysin and excitation at 561 nm and emission at 594 nm for monitoring lipids.

### Reconstitution of proteins into proteoliposomes and assessment of reconstitution efficiency by density gradient centrifugation

For the assessment of proteoliposome diameter using DLS, proteins were reconstituted into LUVs by mixing the detergent-solubilized components and subsequently removing the detergent by dialysis, as previously described (*14*, *22*). The lipid composition consisted of DOPC, DOPS (1,2-dioleoyl-*sn*-glycero-3-phospho-l-serine), and cholesterol (from sheep wool) (all Avanti Polar Lipids) at a DOPC:DOPS:cholesterol molar ratio of 65:10:25 and was prepared by evaporating the organic solvent from the lipids and dissolving the dried lipid film in buffer [300 mM glycine, 10 mM MOPS (pH 7.3), 2 mM MgSO_4_, and 5% *n*-octyl-beta-d-glucopyranoside (Glycon)]. The protein-to-lipid ratios for synaptophysin and VGLUT1 was adjusted to 1:500 and 1:1800, respectively, with a final lipid concentration of 4 mM. Dialysis was performed in 300 mM glycine, 10 mM MOPS-Tris (pH 7.3), and 2 mM MgSO_4_ overnight at 4°C. For control liposomes without protein, the lipids were mixed with corresponding amounts of phosphate-buffered saline (PBS) containing 1% Na-cholate. Proteoliposomes prepared by this method typically result in average diameters ranging from ~150 to 250 nm (measured by DLS).

The reconstitution efficiency of proteoliposomes was determined by Nycodenz density gradient–based centrifugation as previously described (*61*). Briefly, a step gradient consisting of 40% (bottom), 30% (middle), and 0% (top) Nycodenz was prepared with the proteoliposomes mixed into the 40% Nycodenz fraction. Subsequently, the gradient was subjected to centrifugation at 100,000*g* for 90 min, allowing the liposomes to float toward the top of the gradient according to their lower density. The reconstitution efficiency was determined by the fraction of protein colocalizing with the liposome fractions and was assessed by Western blot analysis.

### Measurement of Glut uptake

Glut uptake was performed according to previous publications (*62*, *63*). The uptake was measured with 2 μCi 3H-glutamic acid (Hartmann Analytik GmbH and GE Healthcare) and 10 μg of mouse LP2 per data point in 150 mM K-gluconate, 20 mM 1,4-piperazinediethanesulfonic acid-KOH (pH 7.0), 4 mM EGTA, 2.9 mM MgSO_4_ (corresponding to 1 mM free Mg^2+^), 2 mM ATP, and 50 μM K-glutamate for 15 min at 32°C. Nonspecific uptake was measured in the presence of 30 to 60 μM carbonyl cyanide *p*-trifluoromethoxyphenylhydrazone and was subsequently subtracted from the measurements.

### Electron microscopy of hippocampal neurons

#### Animals

For all procedures, ethical approval was obtained in accordance with the National Act on the Use of Experimental Animals (Germany).

#### Neuronal cell culture

Primary cultures of hippocampal neurons derived from 16-day-old (E16) WT and SYP KO mouse embryos were prepared as described earlier (*64*, *65*). Embryos (littermates) were dissociated and cultured separately with genotyping performed afterward. Dissociated neurons were plated at a density of 2 × 10^4^ cells per well and grown on poly-d-lysine–coated glass coverslips in serum-free neurobasal medium supplemented with B27, 0.5 mM glutamine, penicillin/streptomycin (100 U/ml), and 25 μM Glut at 37°C under 5% CO_2_. At day in vitro 21, neuron monolayers were chemically fixed and processed for morphometric and ultrastructural analyses.

#### Quantitative ultrastructural analysis

WT and SYP KO primary cultures of hippocampal neurons were chemically fixed at day in vitro 21 by 4% paraformaldehyde (Electron Microscopy Sciences, Hatfield, PA) containing 0.1% glutaraldehyde (Electron Microscopy Sciences) in 0.1 M phosphate buffer (pH 7.4). Neurons were postfixed with buffered 0.1% osmium tetroxide (OsO_4_; Electron Microscopy Sciences), stained en bloc with 2% aqueous uranyl acetate (Merck, Darmstadt, Germany), and gradually dehydrated in ethanol. Fixed neurons were infiltrated in hydroxypropyl methacrylate (Sigma-Aldrich Chemie) and Epon-812 resin (Sigma-Aldrich Chemie), embedded on gelatin capsules (Plano), and polymerized for 48 hours at 60°C. After removing the coverslips, ultrathin sections (60 nm) were cut and examined using a Zeiss transmission electron microscope 900 (TEM-900) equipped with a digital camera (Proscan 2K Slow-Scan CCD-Camera, Zeiss, Oberkochen, Germany). For statistical analysis, neurons from three separate culture preparations were examined quantitatively using the shape descriptors in the iTEM Software package (Olympus Soft Imaging Solutions).

#### Fluorescence microscopy

Synaptophysin was detected using a monoclonal antibody (7.2 from SySy) and an Alexa Fluor 488–conjugated secondary antibody (Molecular Probes, Eugene, OR).

### Single-vesicle fusion assay

Purified SVs were frozen and shipped on dry ice and stored at −80°C until use. SVs were thawed and diluted into 200 μl of 150 mM KCl and 20 mM Hepes (pH 7.4). SVs were labeled by drying down 5 μg of Rh-DOPE under N_2_ and then by 1 hour in a vacuum desiccator. SVs were gently vortexed over the dried-down Rh-DOPE until the dye went into solution.

Planar supported bilayers with reconstituted plasma membrane SNAREs [syntaxin-1A and dodecylated SNAP-25 (d-SNAP-25)] were prepared by the Langmuir-Blodgett/vesicle fusion method (*66*). Cleaned quartz slides (by dipping in 3:1 sulfuric acid:hydrogen peroxide for 15 min followed by extensive rinsing in Milli-Q water) were deposited with a lipid monolayer prepared by Langmuir-Blodgett transfer using a Nima 611 Langmuir-Blodgett trough (Nima, Conventry, UK) by applying the lipid mixture of 70:30:3 bPC:Chol:DPS from a chloroform solution that was compressed at a rate of 10 cm^2^ min^−1^ to reach a surface pressure of 32 mN m^−1^ where the slide was rapidly dipped at 68 mm min^−1^ and then slowly withdrawn at 5 mm min^−1^. A computer maintained a constant surface pressure and monitored the transfer of lipids with results in the head groups being down against the hydrophilic substrate. Proteoliposomes with a lipid composition of bPC:bPE:bPS:Chol:PI:PI(4,5)P_2_ (25:25:15:30:4:1) with syntaxin-1A and d-SNAP-25 (lipid:protein of 3000:1), made as previously described (*35*), were incubated above the lipid monolayers at a concentration of 77 μM total lipid for 1 hour in 150 mM KCl and 20 mM Hepes (pH 7.4). Bilayers were then extensively washed by perfusion of 5 ml of 150 mM KCl and 20 mM Hepes (pH 7.4), followed by perfusion of 5 ml of desired buffer for experiments being either 300 mM glycine, 10 mM MOPS (pH 7.3), 10 mM K-glutamate, 4 mM KCl, and 4 mM Mg-ATP for Glut-loaded SVs or 150 mM K-gluconate, 10 mM MOPS (pH 7.3), 10 mM GABA, 10 mM KCl, and 4 mM Mg-ATP for GABA-loaded SVs. Baf was used at a concentration of 100 nM and CCCP at 1 μM.

Bilayers were incubated with 0.5 μM Munc18 and 2 μM complexin-1 for 20 min. SVs were diluted into the desired buffer (5 μl of SVs into 200 μl) and then added to the supported lipid bilayer while keeping Munc18 and complexin-1 constant, with the addition of 0.2 μM Munc13. The supported bilayers were then placed in an incubator, and the temperature was kept constant at 32°C. After 20 min, the bilayers were washed with desired incubation buffer to remove all unbound SVs, Munc18, complexin-1, and Munc13. Bilayers were then washed with 1 ml of desired SV loading buffer and incubated for another 20 min. Bilayers were then moved to a total internal reflection fluorescence microscope with a heated stage. Bilayers were then injected with prewarmed buffer using a syringe pump containing 100 μM Ca^2+^ and a constant concentration of the loading conditions of the desired experiment.

To obtain the kinetic constants *k*_1_ and *k*_2_, first-order kinetics with one (Eq. 1) or two (Eq. 2) components were fitted to the kinetic data using IgorPro (Wavemetrics, Lake Oswego, OR).

$$N\left(t\right)=N_{1}\left[1-e^{-\left(k_{1}t\right)}\right]$$ (1)

$$N\left(t\right)=N_{1}\left[1-e^{-\left(k_{1}t\right)}\right]+N_{2}\left[1-e^{-\left(k_{2}t\right)}\right]$$ (2)

### Immunoenrichments of VGLUT1 and VGAT SVs

Protein A magnetic SureBeads (100 μl; Bio-Rad) were washed three times with PBS with 1% Tween, three times with PBS, and lastly, three times with 150 mM KCl and 20 mM Hepes (pH 7.4). The beads where then incubated with 5 μg of either VGLUT1 or VGAT in 100 μl of 150 mM KCl and 20 mM Hepes (pH 7.4) for 30 min and then washed three times with 0.5 ml of the same buffer. Antibody-bound beads were then incubated with one aliquot of SVs diluted into 100 μl of 150 mM KCl and 20 mM Hepes (pH 7.4). The supernatant of this medium was then taken as either the VGLUT1- or VGAT-depleted SVs. The beads were then incubated with 15 μg of a control peptide to elute the bound SVs to create a VGLUT1- or VGAT-enriched SV.

### Pyrene dye measurements on SVs

The solvatochromic dye PA was a gift of A. Klymchenko (Strasbourg, France) (*23*, *67*). SVs were labeled with the PA dye in the same manner as the Rh-DOPE dye. SVs were then diluted by adding 1 μl in 400 μl. SVs were then incubated on glass coverslips coated with poly-d-lysine (0.1 mg/ml) for 30 min. The desired buffer for loading SVs (see the “Single-vesicle fusion assay” section) was added, and the coverslips were incubated at 32°C. SVs were imaged using an LSM880 with a spectral detector scanning the emission wavelengths from 420 to 650 nm.
