# Supplementary material for: Synaptophysin accelerates synaptic vesicle fusion by expanding the membrane upon neurotransmitter loading
Source: Sci Adv. 2025 Apr 23;11(17):eads4661. doi: 10.1126/sciadv.ads4661 (PMC12017324; doi:10.1126/sciadv.ads4661)
Supplement: Supplementary file 1 — Figs. S1 to S4 [file sciadv.ads4661_sm.pdf]

Science Advances
AAAS

Supplementary Materials for

Synaptophysin accelerates synaptic vesicle fusion by expanding the membrane upon neurotransmitter loading

Julia Preobraschenski *et al.*

Corresponding author: Reinhard Jahn, rjahn@mpinat.mpg.de;
Julia Preobraschenski, julia.preobraschenski@med.uni-goettingen.de

Sci. Adv. **11**, eads4661 (2025)
DOI: 10.1126/sciadv.ads4661

This PDF file includes:

Figs. S1 to S4

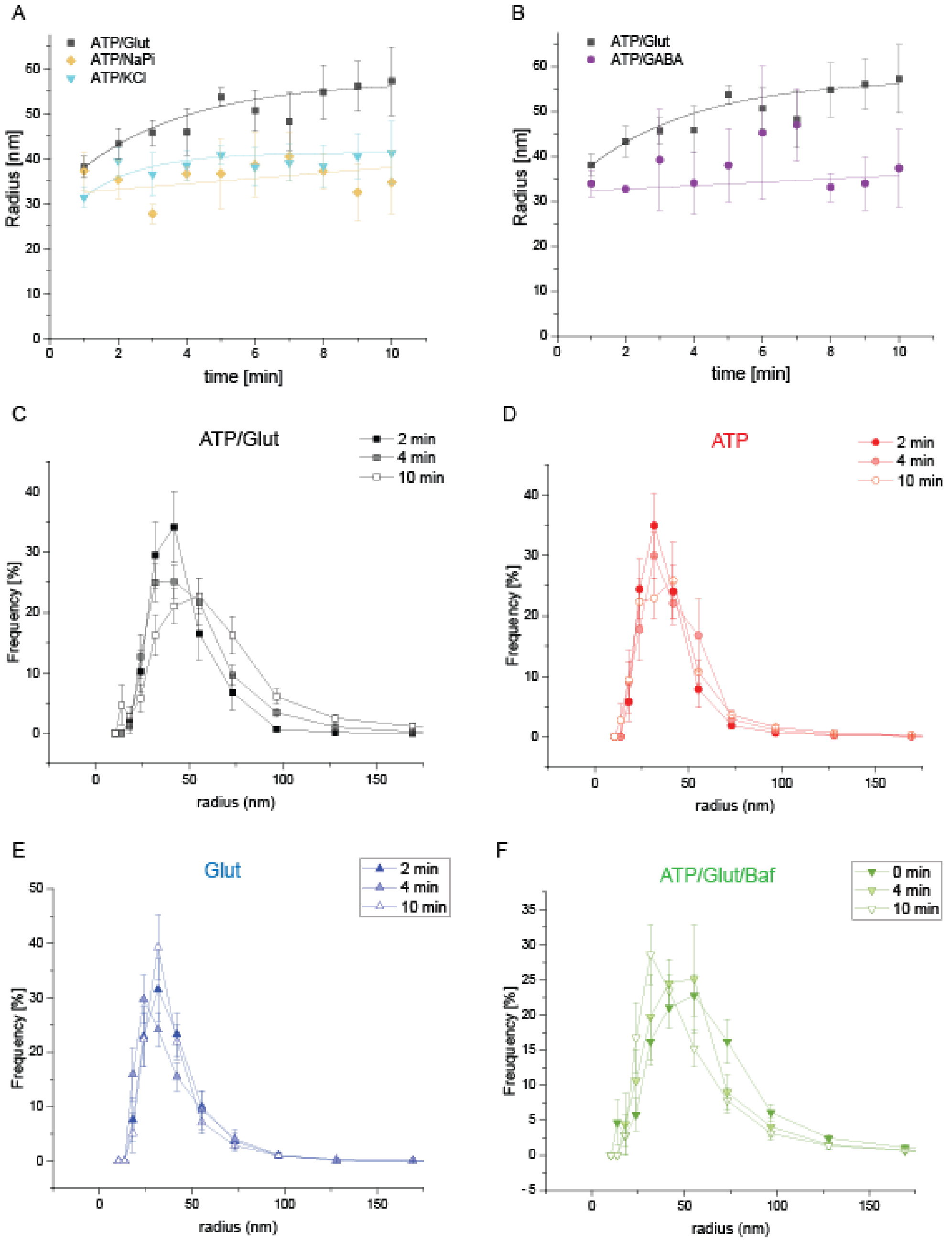

Fig. S1.

Histograms of size changes of SVs during incubation with ATP and glutamate, GABA, chloride, and inorganic phosphate, measured by DLS. (A) Average time-dependent size of SVs at glutamate uptake conditions (black squares), 10 mM KCl and ATP (cyan triangles), 10 mM K-phosphate and ATP (yellow diamonds) and10 mM GABA and 4 mM ATP (purple circles) in uptake buffer. The reaction was started by the addition of ATP and respective substrate. (B) to (E): Time-dependent size distribution of SVs measured by DLS at (B) glutamate uptake conditions (ATP/Glut; black line),and exclusively in presence of (C) ATP (ATP; red line) or (D) glutamate (Glut; blue line) and (E) after addition of Baf to glutamate-filled SVs after 2 (0 (F)), 4 and10 min of incubation at 37 °C. n (A) = 2-6; (B)-(D) = 6; (E) = 4.

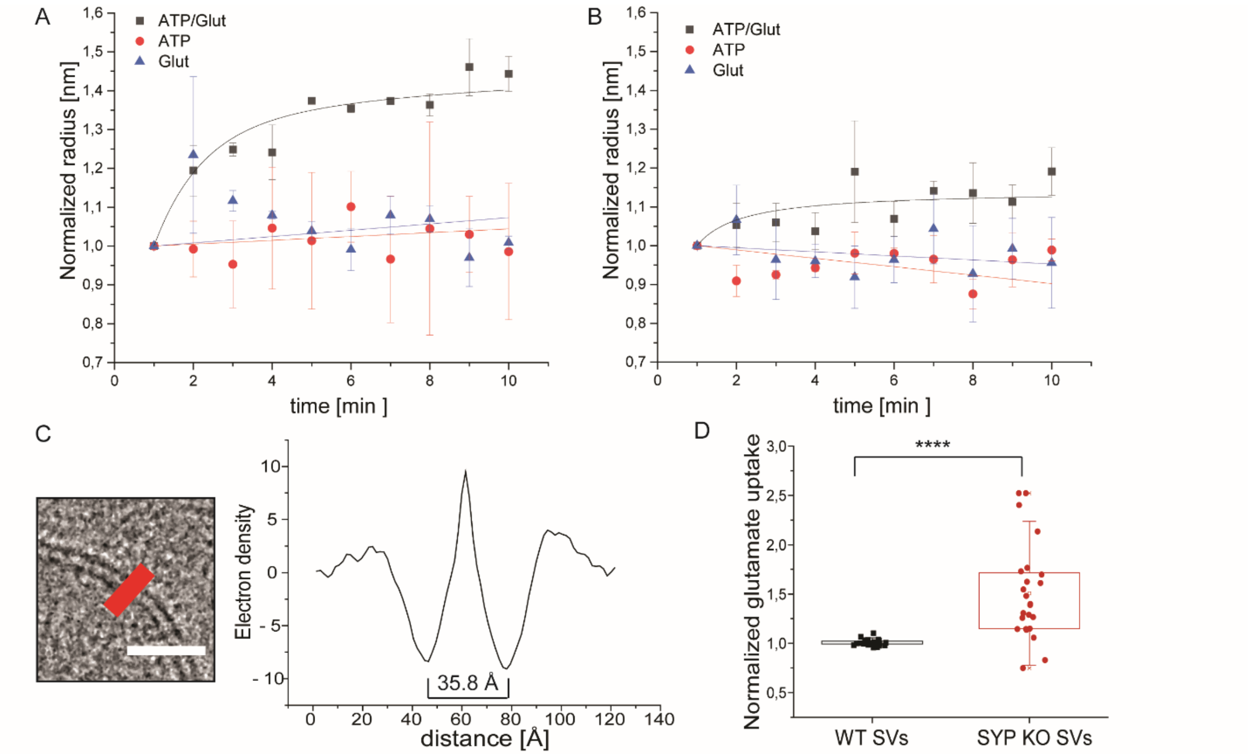

Fig. S2.

SYP is involved in SV membrane expansion. Control experiments for the difference in time-dependent expansion of WT (A) and SYP KO (B) SVs during glutamate uptake (related to Fig. 2B, with the curves of 2B shown as reference: ATP/Glut, green squares). ATP only (red circles) and glutamate only (blue triangles). Size changes were measured by DLS. (C) Membrane thickness of SVs measured by line scans across the lipid bilayer in cryo-EM images (indicated by red bar in the left panel). Thickness is determined by the distance between the two minima corresponding to lipid headgroups of the bilayer. Scale bar in the left panel corresponds to 20 nm. (D) Comparison of glutamate uptake by WT and SYP KO SVs reveals slightly increased uptake by SYP KO SVs that is highly variable. The data in (D) were analyzed using a two-tailed paired t-test, ****$p < 0.0001$. n (A) = 2; (B) = 3; (D) = 23.

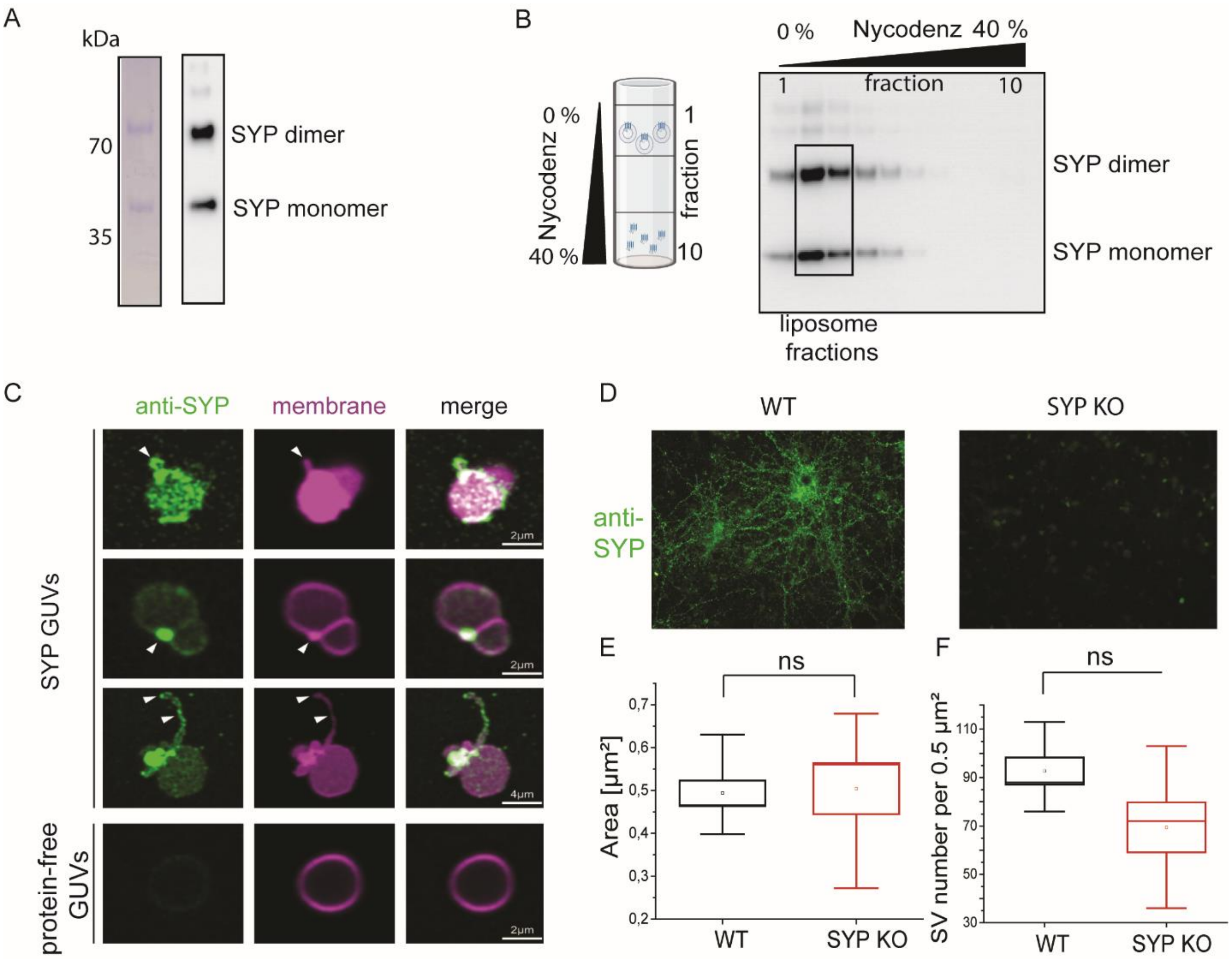

Fig. S3.

Synaptophysin induces membrane curvature. (A) Coomassie-Blue-stained SDS-polyacrylamide gel and immunoblot for synaptophysin of the purified protein (note that synaptophysin has a tendency to dimerize upon heating in SDS). (B) Individual fractions from a density gradient with synaptophysin-reconstituted liposomes (related to Fig. 3F) showing efficient insertion of the protein into liposomes as demonstrated by strong co-localization of synaptophysin with the liposome fractions. Immunoblot for synaptophysin. Schematic on the left shows hypothetical localization of liposome-incorporated and free protein on the density gradient. (C) Representative images of GUVs prepared from SUVs containing synaptophysin, showing a preferential localization of synaptophysin in highly curved regions and small protrusions. Synaptophysin was labelled with antiSYP antibody, displayed in green, and lipids were labelled with DOPE Texas Red, displayed in purple. (D) Representative images of WT (left panel) and SYP KO (right panel) hippocampal neurons labelled with a fluorescent SYP-antibody (69.1, Synaptic Systems). (E) and (F): Characterization of mouse primary hippocampal neurons from WT and SYP KO mice: Box plots representing the average (I) synaptic bouton area and (J) number of SVs per 0.5 µm² in WT (black box) and SYP KO (red box) showing no difference in the area but a reduced number of SVs in SYP KO boutons compared to WT. Quantification based on micrographs acquired by EM. The box represents data between 25 and 75 %, the whiskers show data between 10 and 90 %. The dot within the box indicates the mean value. The data in (E) and

(F) were analyzed using a two-tailed paired t-test, ns (not significant): (E) p = 0.88; (F) p = 0.07. n (E) = 7; (F) = 7.

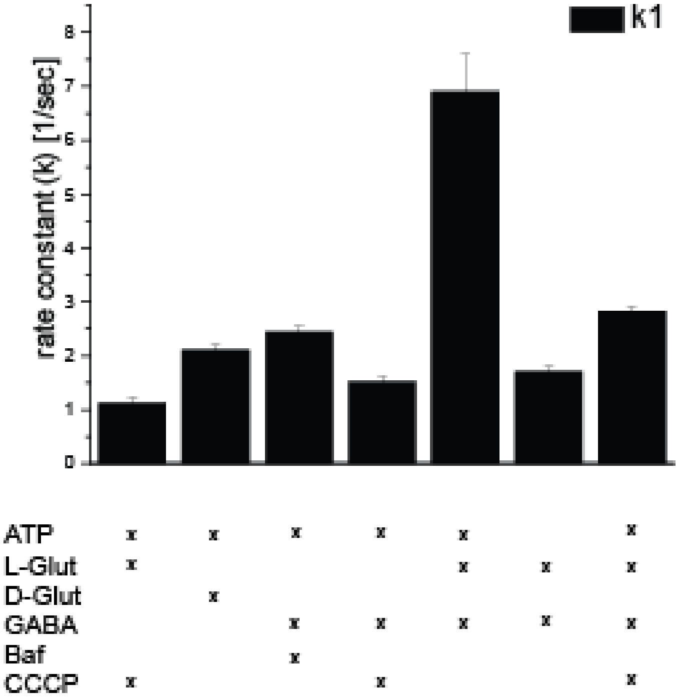

Fig. S4.

Rate constants and total fusion of filled and empty SVs. Rate constants of SV fusion at additional conditions related to Fig. 4C determined with a one-component (k1) fit. n = 5-7.
